# Routine clinical mutation profiling using next generation sequencing and a customized gene panel improves diagnostic precision in myeloid neoplasms

**DOI:** 10.18632/oncotarget.8310

**Published:** 2016-03-23

**Authors:** Stephan Bartels, Elisa Schipper, Britta Hasemeier, Hans Kreipe, Ulrich Lehmann

**Affiliations:** ^1^ Institute of Pathology, Medizinische Hochschule Hannover, Hannover, Germany

**Keywords:** myeloid neoplasms, next-generation sequencing, FFPE, MDS, hematopathology, Pathology Section

## Abstract

Microscopic examination of myelodysplastic syndromes (MDS) and myelodysplastic-myeloproliferative neoplasms (MDS/MPN) may be challenging because morphological features can overlap with those of reactive states. Demonstration of clonal hematopoiesis provides a diagnostic clue and has become possible by comprehensive mutation profiling of a number of frequently mutated genes, some of them with large coding regions.

To emphasize the potential benefit of NGS in hematopathology we present sequencing results from routinely processed formalin-fixed and paraffin-embedded (FFPE) bone marrow trephines (*n* = 192). A customized amplicon-based gene panel including 23 genes frequently mutated in myeloid neoplasms was established and implemented. Thereby, 629,691 reads per sample (range 179,847–1,460,412) and a mean coverage of 2,702 (range 707–6,327) could be obtained, which are sufficient for comprehensive mutational profiling. Seven samples failed in sequencing (3.6%). In 185 samples we found in total 269 pathogenic variants (mean 1.4 variants per patient, range 0-5), 125 Patients exhibit at least one pathogenic mutation (67.6%). Variants show allele frequencies ranging from 6.7% up to 95.7%. Most frequently mutated genes were TET2 (28.7%), SRSF2 (19.5%), ASXL1 (8.6%) and U2AF1 (8.1%). The mutation profiling increases the diagnostic precision and adds prognostic information.

## INTRODUCTION

Targeted cancer therapies have been developed for many types of tumors and the necessity for precise analysis of a number of marker genes for every single patient will rise in the next years [1–3]. In the field of hematopathology analyzing the mutational status of specific driver genes can be helpful to discriminate different disease subtypes. This is especially important if morphological examination or clinical presentation remains inconclusive [4–6]. Taken together, mutational profiling could improve the diagnostic accuracy, contribute to more precise risk stratification and reveal new therapeutic options [7, 8].

Myeloid neoplasms show a complex genetic basis, with 50-60 assumed driver genes for myelodysplastic syndromes alone [9]. Additionally, many important marker genes exhibit large coding sequences (for example ASXL1, DNMT3A, TET2 and RUNX1), which would be very time-consuming and expensive for Sanger sequencing. Furthermore, conventional sequencing does not provide sufficient sensitivity, especially for the analysis of minor sub-clones. High-throughput next-generation sequencing (NGS) allows rapid analysis of patients in a constantly increasing number of molecular markers [10,11]. Research studies performed with NGS techniques and patient cohorts of different disease entities already contributed considerably to a better understanding of the development, progress and therapy of the myeloid neoplasms [12–14]. Therefore, we designed and established a customized amplicon-based NGS panel including 23 genes frequently mutated in MDS and MPN.

Our results show that nearly all of the analyzed genes are affected by pathogenic mutations in routine patient samples. Many patient samples show more than one mutation and the specific combination of the affected genes can be helpful for diagnosis. For example, concomitant mutations of TET2 and SRSF2 are very frequent in chronic myelomonocytic leukemia (CMML) [15–17]. The preferred specimens are fixed, decalcified and embedded (FFPE) bone marrow trephines, because this sample type best provides morphological details of the bone marrow and the feasibility of NGS could be shown in different studies [18–21].

In addition, we systematically reviewed the scientific literature and validated quality parameters for NGS mutation profiling of FFPE samples in order to reduce the risk of false-positive variant reporting.

## RESULTS

### Performance characteristics of the MDS/MPN-Panel

For the technical implementation of the MDS/MPN-Panel 24 FFPE patient samples with known pathogenic mutations from the years 2002 till 2015 were sequenced with 8 samples per run. Mean values and ranges of the most important technical parameters are shown in Table 1. One sample failed in sequencing (4.2%). Table S1 compares variant calls from two follow-up biopsies of a patient with known minor JAK2 p.V617F clone of approx. 5% variant allele frequency.

Validation of variant calling of the MDS/MPN panel was performed with the commercially available QMRS reference standard HD200 from Horizon Discovery™. Thereby, the following mutations could be detected reproducibly with our new panel (allele frequencies as specified by the manufacturer): BRAF p.V600E (10.5%), KIT p.D816V (10.0%), KRAS p.G12D (6.0%), p.G13D (15.0%) & NRAS p.Q61K (12.5%). Table S2 shows the results for the HD200 control from two different runs, one with approx. 30% less reads than the average in the implementation phase and one sample with approx. 30% more reads.

**Table 1:** Technical performance of *n* = 23 FFPE patient samples for implementation of the MDS/MPN-Panel

	Mean (Range)
Mapped reads	721,980 (351,690 – 1,018,128)
Mean coverage per base	3,110 (1,469 – 4,416)
Reads on target sequence	97.8% (95.3 – 99.5)
Read uniformity	95.8% (87.6 – 98.3)
Mean read length	125 bp. (117 – 141)

Mean values and range of sequencing parameters are indicated.

### Sequencing performance of the MDS/MPN-Panel in routine diagnostics

In total, 192 FFPE patient samples were analyzed, with 8 patient samples run in parallel on a 318 v2 chip. Only seven samples failed in sequencing (3.7%). The remaining 185 samples exhibit more than 600,000 reads per sample and a mean coverage per base of more than 2,700x (Table 2), which ensures proper mutation profiling. Some samples with low DNA quality performed much worse than the average. Table S3 shows all sequencing results from all analyzed patient samples including DNA concentration and quantification of the three library pools.

**Table 2:** Sequencing performance of *n* = 185 FFPE patient samples

	Mean (Range)
Mapped reads	629,691 (179,847 – 1,460,412)
Mean coverage per base	2,702 (707 – 6,327)
Reads on target sequence	97.6% (86.6 – 99.5)
Read uniformity	92.5% (55.3 – 98.7)
Mean read length	122.9 bp. (97 – 141)

Mean values and range of sequencing parameters are indicated.

**Figure 1:**
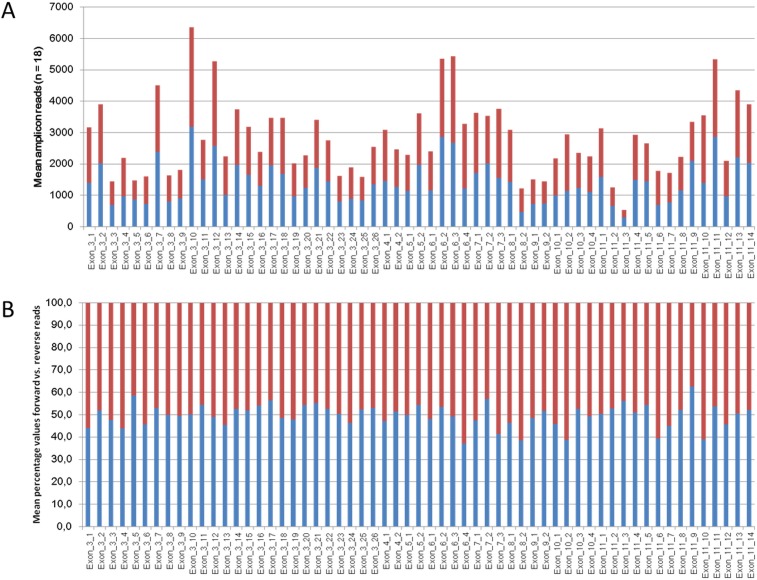
**A.** Mean amplicon coverage of the 59 amplicons of TET2. Shown are the coverage of the forward reads in blue bars and the coverage of the reverse reads in red bars. Values of 18 samples from 5 independent analyses were averaged. **B.** Percentages of the distribution from forward and reverse reads of the 59 amplicons of TET2. Values of 18 samples from 5 independent runs were averaged.

To illustrate amplicon coverage uniformity Figure 1 shows the mean forward and reverse amplicon coverage for TET2 which is covered by 59 amplicons. It can be noticed that only one amplicon (Exon_11_3, Figure 1A) exhibits mean amplicon coverage below 1000 reads. Figure 1B illustrates the distribution of forward and reverse reads among the 59 amplicons covering TET2. The highest imbalance is shown by amplicon Exon_6_4 with 37.1% forward and 62.9% reverse reads. Table S4 summarizes the mean amplicon coverage of all 243 amplicons as well as mean counts for forward and reverse reads from 18 patient samples which were sequenced in five independent runs.

### Mutation profiling in myeloid neoplasms

Overall 269 pathogenic mutations could be found in 125 out of 185 analyzed patients (1.4 variants per patient). 67.6% of the patients harbor at last one pathogenic mutation (Range 0 - 5). 22 out of the 23 genes in the MDS/MPN-Panel show at last one patient with a variant, only the KIT gene (Exon 8, 10, 11 and 17) shows a wild-type sequence in all 185 patients. The ten most frequently mutated genes are depicted in Table 3. Interestingly, from the 53 TET2-mutated patients 24 show more than one pathogenic mutation in TET2, one patient even three. Out of these 24 patients with more than one TET2 variants 15 patients exhibit a CMML disease (remaining 9 patients show 2^nd^ AML, RCMD, MDS-unclassifiable, and MDS/MPN-unclassifiable, respectively). Additionally, NRAS and EZH2 show two variants in two patients and CSF3R show two variants in one patient, respectively. Table S5 shows all 269 variants which could be detected in the 185 patients, including allele frequencies, sequencing depth, and quality of the variant call. Furthermore, the morphological diagnoses of the bone marrow specimens are listed.

**Table 3:** The ten most frequently mutated genes in our patient cohort

Gene	No. of mutations	No. of mutated patients	% mutated patients (*n* = 185)
*TET2*	78	53	28.7%
*SRSF2*	36	36	19.5%
*ASXL1*	16	16	8.6%
*U2AF1*	15	15	8.1%
*TP53*	14	14	7.6%
*JAK2*	13	13	7.0%
*SETBP1*	12	12	6.5%
*RUNX1*	11	11	5.9%
*DNMT3A*	10	10	5.4%
*EZH2*	11	9	4.9%

*TET2* show more than one variant in 24 patients. *NRAS* and *EZH2* show two mutations in two patients. *CSF3R* show more than one variant in one patient. (*NRAS* and *CSF3R* are not among the ten most frequently mutated genes).

**Figure 2:**
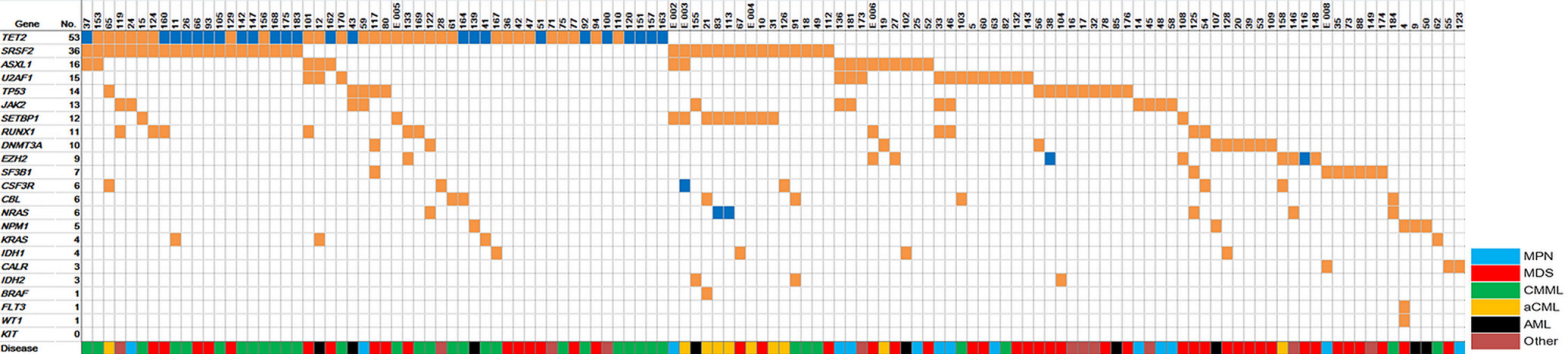
Distribution of mutations among 125 patients with detectable pathogenic variants “No.” indicates the number of patients with detectable pathogenic somatic mutations. The color-coded disease type represents the suspected classification of the specific myeloid neoplasm after morphological evaluation and before comprehensive mutational profiling. If a patient shows two somatic mutations the box is colored in dark blue.

In Figure 2 the distribution of the detected variants among the 185 patient samples is illustrated. Together with Table S5 known associations between mutations and disease subtypes can be noticed. The combination of TET2 and SRSF2 mutated, for instance, is frequently found in CMML, whereas the combination of SRSF2 and SETBP1 mutation is frequent in aCML.

**Figure 3:**
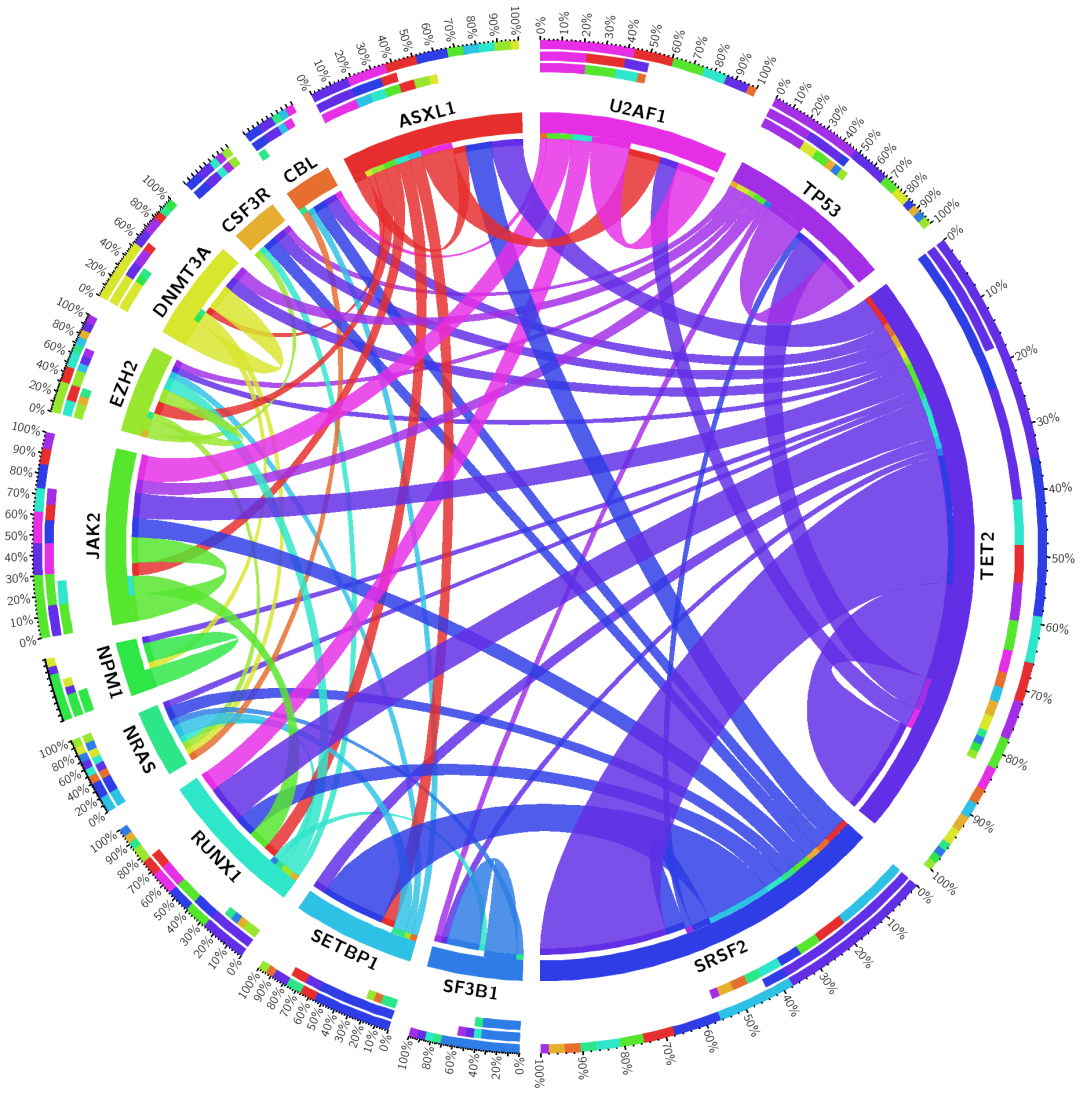
Circos diagram revealing concomitantly mutated genes Shown are only those genes which were found to be mutated in at least five patients (out of 185 patients).

Figure 3 gives an overview of the concomitantly mutated genes in the study cohort. TET2 and SRSF2 show co-mutation with nearly all other frequently mutated genes, including each other. SETBP1 is nearly always co-mutated with SRSF2 and together these two genes are typically found in aCML. U2AF1, SF3B1, and DNMT3A are more often the only detectable pathogenic variants. These “single mutated cases” mostly show a MDS disease phenotype (see Figure 2), whereas a NPM1 mutation is highly specific for 2^nd^ AML. No co-mutations of two splice factor genes were detected in the 185 patients (Figure 3). Other classes of genes do not show this strict mutual exclusivity. For example epigenetic genes (DNMT3A and TET2) and genes encoding histone modifiers (ASXL1 and EZH2) could be found mutated concomitantly.

### Age distribution of patients with and without mutations

TET2 is the most frequently mutated gene in our patient cohort and is known to be frequently mutated in elderly persons without myeloid neoplasms. Table 4 shows the age distribution of mutated and non-mutated patients, as well as TET2-mutated patients. Patients harboring one or more mutations are significantly older than non-mutated patients (72.0 vs. 59.8 years, p<0.0001, independent t-test). This is also true for TET2 mutated patients, who show a mean age of 76.6 years (76.6 vs. 59.8 years, p<0.0001, independent t-test).

**Table 4:** Mean values of patient age in our cohort

	Total	Non-mutated	Mutated	*TET2* mutated	*TET2* one mutation	*TET2* >one mutation
Number of patients (%)	185 (100.0%)	60 (32.4%)	125 (67.6%)	53	28	25
Mean age	68.1	59,8	72.0	76.6	74.8	78.8
Median age	72	63	74	78	77	80
SD	14.6	17.7	10.8	7.9	8.9	5.8
Range	14-91	14-87	36-91	55-91	55-91	67-88

### Diagnostic relevance of mutation profiling

For 87 out of the 185 patients microscopic examination in conjunction with the available clinical data did not provide a definite diagnosis (see Table S5).

In 44 out of these 87 patients (50.6%) one or more pathogenic mutations in the 23 genes analyzed could be detected. Table 5 summarizes the different suspected subtypes of myeloid neoplasms after morphological evaluation and depicts how many patients exhibit pathogenic variants.

**Table 5:** Patients with ambiguous morphological findings, which are analyzed with the MDS/MPN-Panel to precise the diagnosis

Suspected myeloid neoplasm	No. patients with detectable variant
MDS (*n* = 55)	24 (43.6%)
MPN (*n* = 9)	4 (44.4%)
CMML (*n* = 18)	14 (77.8%)
Other* (*n* = 5)	2 (40.0%)

Other* include aCML, CML and MDS/MPN-U

### Quality parameters in targeted resequencing

Based on our experience with amplicon-based targeted re-sequencing and the analysis of FFPE tissue specimens we highly recommend the definition of strict quality parameters for mutation profiling. Many studies which are dealing with the establishment of NGS technologies do not specify any quality parameters for the assessment of variant calls [19,22–28]. Unfortunately, this makes the comparison of results from different studies often difficult or even impossible. Nevertheless, we found a series of studies which indicated quality parameters for variant calling (listed in Table 6).

**Table 6:** Suggested quality parameters for variant calls in other implementation studies for NGS

Source	Total reads per sample	Coverage for variant calls	Allele frequency	Variant calls in for and rev strands
[1]	−	>500x	>5%	−
[11]	−	>50x	−	−
[44]	−	>80x	>20%	+
[45]	>300,000	>500x	>10%	−
[46]	>100,000	>500x	−	−

“–” indicates no statement about the specific parameter in this study.

Table 7 summarizes our recommendations for the quality parameters for variant calls for mutation profiling in FFPE samples.

**Table 7:** Minimum quality parameters suggested for mutation profiling in molecular diagnostic

Quality parameter	Explanation
5% variant allele frequency of the variant call	Probability of validation with other sequencing technologies
Forward and reverse strands show the variant	Variants detected only in one strand are high likely artifacts
>500x coverage at the variant position	Enough sequencing reads for proper mutation calling
PHRED-scaled quality of 100	Low probability of incorrect variant call

## DISCUSSION

In this study we designed a panel comprised of 23 genes frequently mutated in myeloid neoplasms. The overall sequencing performance of the 185 patient samples under study demonstrates high quality values for total reads, reads mapped on target sequence, uniformity and mean read lengths, respectively (Tables 2 and S3). Also the sequencing performance of all individual amplicons reached a sufficient number of total reads as well as a balanced forward and reverse read ratio (Figure 1A and 1B, Table S4).

DNA isolation, library preparation, massive parallel sequencing, and data evaluation can be accomplished in 5-7 working days. The reagent costs depend very much on the number of samples processed in parallel and per month as well as on the overall throughput of the institution. Prices may also vary between countries and continents. For our institution we calculate with reagent costs of approximately 300 £ per sample. This sum does not cover investments, maintenance and staff.

### Diagnostic relevance of routine mutation profiling

Recent studies describe the detection of somatic mutations in disease associated genes in elderly individuals without clinical evidence for myeloid neoplasms [29–32]. These authors found somatic mutations with low allele frequencies in up to 10% of persons over 65 years, and even in nearly 20% in persons older than 90 years [31, 32]. The most frequently affected genes are DNMT3A, ASXL1 and TET2. This led to the assumption that these genes play an initiating role in the age-associated hematologic cancers [29]. In a previous study we could show that PMF patients which underwent a disease progress in follow-up biopsies already showed the SRSF2 mutated clone in similar allele frequencies in the initial biopsy [33]. However, similar to NPM1 and FLT3 mutations in acute myeloid leukemia (AML), SRSF2 mutations seem to occur later in the development of myeloid neoplasms than TET2 mutations.

It is known that pathogenic mutations can occur in individuals that will never develop a disease [4]. Consequently, the detection of a cancer-associated mutation alone does not justify the diagnosis of hematologic malignancies [32]. The results and frequencies described in our study are not directly comparable because we performed molecular analyses only in those individuals for whom the morphologic examination of the bone marrow trephine raised the suspicion of a myeloid disorder. For example, a suspected CMML diagnosis can be confirmed by the detection of concomitant SRSF2 and TET2 mutation. Since concomitant SRSF2 and TET2 mutations can also be found to a certain degree in other MDS subtypes [13, 34], the mutation profile does not define a diagnosis on its own and has to be evaluated in the context of the clinical findings (e.g., sustained monocytosis ≥1000 μl^−1^, ≥10% monocytes) and morphological features (e.g., dysplastic features in at least one cell lineage).

Especially the distinction between an incipient myeloid neoplasm and other reasons for dysplastic changes of bone marrow cells (e.g. infections, nutritional deficiencies or cytotoxic damages through chemotherapeutic agents) can be supported by mutation profiling. Almost all MDS cases exhibit at least one mutation [5]. Consequently, the detection of a gene mutation in patients which show clinical features of an incipient MDS strongly supports the diagnosis. We demonstrate that 44 out of 87 patients (50.6%) with clinically and morphologically ambiguous changes in hematopoiesis show a mutation in at least one gene with disease relevance in myeloid neoplasms (Table 4 and Table S5). Considering the morphologic examination and the somatic mutations together, these individuals are highly likely to have an incipient myeloid neoplasm. However, in light of the findings that MDS-associated somatic mutations can be found to a certain degree in healthy elderly people [4, 35, 36] a confirmatory follow-up biopsy is always preferable for a concluding diagnosis.

In conclusion, comprehensive molecular profiling can support the discrimination of distinct myeloid neoplasms as well as an incipient myeloid neoplasm from other reasons of conspicuous clinical findings in the bone marrow.

If a variant call failed to fulfill the criteria summarized in Table 7, we strongly recommend the validation of the called variant with a second sequencing method or repetition of the NGS analyzes. Routine validation of variants with low allele frequencies as well as the regular analysis of reference material with defined mutation status along the routine samples is strongly recommended in order to maintain a constant high quality of the molecular diagnostics.

If only one strand shows a variant, it is high likely that the variant represent a PCR or sequencing artifact. A limit of 500x total sequencing depth and a PHRED-scaled quality above 100 for the analysis of FFPE samples are suitable quality criteria based on our experience. Nevertheless, also sequencing artifacts due to formalin fixation can fulfil these criteria. However, these artifacts could successful be removed by treatment of the input DNA with Uracil-DNA-Glycosylase (UNG) [21, 37].

In conclusion, comprehensive mutation profiling by NGS with customized gene panel represent a valuable tool to support morphologic examination of the bone marrow in diagnostics of myeloid neoplasms, improves overall diagnostic accuracy and adds important prognostic information.

## MATERIALS AND METHODS

### Patient samples

192 cases from the routine diagnostic procedures of the Institute of Pathology were analyzed between February and December 2015. Tissue specimens are fixed, decalcified and embedded (FFPE) bone marrow trephines. The only selection criterion was the amount of DNA, which was available for library preparation. The patient samples represent a spectrum of myeloid neoplasms, mostly myelodysplastic syndromes (MDS) and myelodysplastic/myeloproliferative neoplasms (MDS/MPN) cases, following the classification system of the World Health Organization [38]. The study design is following the guidelines of the local ethics committee (“Ethics committee of the Medical School Hannover/Ethik-Kommission der Medizinischen Hochschule Hannover”, head: Prof. Dr. Tröger).

### Tissue processing

Bone marrow trephines are fixed for at least 24h in bone marrow fixative (64% methanol (v/v, J.T.Baker^®^), 4.48M formaldehyde (Merck), 1.6mM sodium hydrogen phosphate pH 7.4, 7.4mM glucose). Fixed trephines were decalcified in the ultrasonic decalcifying automat USE 33 (Medite) by incubation at 16°C overnight in EDTA solution (270mM Tris-HCl (Merck), 270mM EDTA (Merck), pH 7.4). Subsequently, trephines are embedded in paraffin (62°C).

### DNA-Isolation

From the FFPE blocks 15 μm thick sections were collected. Up to 5 sections, depending on the size of the biopsy were extracted with DNeasy Blood and Tissue Kit (Qiagen, Hilden, Germany). DNA quantification was performed using the Qubit 2.0 Fluorometer with Qubit dsDNA HS (High Sensitivity) Assay Kit (Life Technologies, Carlsbad, CA, USA). Mean DNA concentration was 25.6 ng/μl.

### Customized MDS/MPN-Panel

The customized MDS/MPN-Panel comprises 243 amplicons from 23 genes which are frequently mutated in myeloid neoplasms (see Table S6). The panel consists of 3 primer pools; each requires a minimum of 10 ng DNA input material. Amplicon lengths are between 68 and 185 bp (mean 124 bp) and cover 27 kb in total. Design of the panel primers were performed with the Ion Ampliseq Designer (pipeline version 4.2). MPL Exon 10 is missing in the gene panel, because primers for MPL failed in the multiplex-PCR for library preparation nearly completely. MPL sequencing was performed separately with Pyrosequencing^™^ as described [39]. Table S7 contains all primer sequences for the 243 amplicons.

### Semiconductor-based targeted resequencing

Library preparation was performed with Ion AmpliSeq Library Kit 2.0. Quantification of prepared libraries was conducted by qPCR using the Ion Library Quantification Kit. For template preparation using the Ion OneTouch 2 instrument 8 patient samples were pooled (100 pM each). Sequencing was performed with Ion PGM Hi-Q Kit v2 and using 318 v2 Chips.

### Bioinformatics

Analyses of sequencing raw data were performed with Torrent server software (Version 4.2.1), IGV-Browser (Version 2.3.34) and Cartagenia Bench Lab NGS software (Version 4.0). Single nucleotide variants with an allele frequency <2% and complex mutations with an allele frequency <5%, and a quality score (Phred-scaled probability of incorrect calls) below 100 were excluded from further analyses. Variant positions have also to be covered by at least 500 reads.

Variants are considered as SNPs if at least two of the following criteria are fulfilled: population frequency of the variant in the 1000 Genomes database [40] with a minimum frequency of 1% and a minimum count of 10; population frequency in the ESP6500 database (NHLBI Grand Opportunity Exome Sequencing Project) with a minimum frequency of 1% and a minimum count of 10; validated SNP in the dbSNP database (National Center for Biotechnology Information (NCBI)) with a population frequency of 0.5% and a minimum count of 10.

Variants are considered as pathogenic with help of the following criteria: the variant is a known hotspot mutation well described in the scientific literature; the variant is listed in the ClinVar database (NCBI) and considered as pathogenic. Furthermore, the functional effect of the variant is predicted with MutationTaster [41] and Variant Effect Predictor software [42]. Novel unknown variants which result in a frameshift or a nonsense mutation of the protein coding region are also considered “pathogenic”.

The presence of a specific variant in the COSMIC database alone (“Catalogue of somatic mutations in cancer”) [43], does not allow to consider the variant as pathogenic, because also well-known SNPs (TP53 p.P72R, KIT p.D185H, and TET2 p.I1762V, for instance) are listed in the database.

## SUPPLEMENTARY MATERIAL TABLES




