# Supplementary material for: Routine clinical mutation profiling using next generation sequencing and a customized gene panel improves diagnostic precision in myeloid neoplasms
Source: Oncotarget. 2016 Mar 23;7(21):30084–93. doi: 10.18632/oncotarget.8310 (PMC5058665; doi:10.18632/oncotarget.8310)
Supplement: Supplementary file 1 [file oncotarget-07-30084-s001.pdf]

Routine clinical mutation profiling using next generation sequencing and a customized gene panel improves diagnostic precision in myeloid neoplasms

Supplementary Material

Table S1: Comparison of variant calls from two follow-up biopsies sequenced in independent runs. Follow-up biopsies originate from a patient with known *JAK2* V617F mutation with minimal allele frequency.

					Sample 04/2002							Sample 11/2002						
Chrom	Position	Ref	Variant	Region Name	Frequency	Quality	Original Coverage	Allele Cov	Allele Cov+	Allele Cov-	Frequency	Quality	Original Coverage	Allele Cov	Allele Cov+	Allele Cov-
chr2	25458379	G	A	AMPL7158937902	100.0	31811.9	2954	1994	1105	889	100.0	31790.7	2916	1993	1069	924
chr2	25458546	C	T	AMPL7153220281	100.0	31887.2	3139	1995	1043	952	100.0	31814.9	3098	1994	1052	942
chr2	25463483	G	A	AMPL7158937916	51.0	10480.7	2180	1019	520	499	47.7	9444.2	2390	954	492	462
chr2	25466888	G	T	AMPL7153037750	100.0	31983.3	3084	1994	844	1150	100.0	31897.4	2895	1990	856	1134
chr2	25468903	C	T	AMPL7157676388	4.4	67.3	2444	88	60	28	5.9	169.6	1967	117	73	44
chr4	106155637	C	T	AMPLP227749409	47.5	5864.0	1249	593	295	298	43.6	6329.7	1552	677	298	379
chr4	106196092	C	T	TET2_83.1.251757	100.0	30043.9	1892	1886	939	947	100.0	31738.3	2333	1991	963	1028
chr4	106196213	C	T	TET2_83.1.9577	49.7	2986.0	603	300	166	134	39.0	2759.9	798	312	169	143
chr4	106196951	A	G	AMPLP227360575	52.8	4149.4	756	400	204	196	50.2	5845.6	1139	574	291	283
chr4	106197561	T	A	TET2_83.3.124254	5.3	119.9	4388	105	63	42	4.9	95.8	3976	98	60	38
chr5	170837457	A	G	NPM1_95.96219	59.4	1319.8	195	117	97	20	44.4	1293.0	312	139	98	41
chr7	148508833	A	G	AMPL7155533243	97.9	26530.7	2466	1955	1030	925	96.0	27707.4	3513	1920	1023	897
chr9	5073770	G	T	AMPLP224028005	4.7	52.3	1097	52	4	48	7.1	218.5	1554	111	23	88
chr11	32417945	T	C	WT1_142.70263	49.2	9900.1	2337	982	551	431	51.8	10738.3	2304	1035	527	508
chr11	119149134	TG	GT	CBL_157.8447	5.4	81.1	1165	63	38	25	9.0	239.4	667	115	100	15
chr17	7578115	T	C	TP53_8.884088	100.0	31340.3	2317	1978	911	1067	100.0	31809.2	2335	1991	935	1056
chr17	7578645	C	T	TP53_10.1.470	100.0	26084.3	1631	1635	665	970	100.0	29414.0	1847	1848	802	1046
chr17	7579472	G	C	TP53_11.1324631	50.2	10036.5	1596	986	410	576	51.3	10368.1	1843	1005	480	525
chr19	13054781	G	T	AMPL7158937925	100.0	31969.0	2470	1998	955	1043	99.9	31830.4	2560	1993	931	1062
chr20	31022959	T	C	ASXL1_235.2.155437	100.0	31564.8	3066	1985	892	1093	100.0	31789.5	2492	1992	875	1117

Table S2: Technical performance of the HD200 control from Horizon Discovery™ DNA sequenced with our MDS/MPN-Panel. Results from two different runs are shown. In total five pathogenic mutations of the control DNA are targeted from the panel.

	**Run 1**			**Run 2**		
**Mapped Reads**	518815			965600		
**Mean Depth**	2325			4282		
**On Target**	98,30%			98,31%		
**Uniformity**	98,70%			94,86%		
	**Frequency**	**Reads**	**Quality**	**Frequency**	**Reads**	**Quality**
***NRAS* p.Q61K**	11,40%	2175	782,2	11,20%	8812	767,2
***KIT* p.D816V**	8,40%	1506	309,8	8,20%	1462	288,1
***BRAF* p.V600E**	12,70%	3734	983,0	11,80%	3363	849,3
***KRAS* p.G12D**	14,00%	1599	951,8	12,00%	1412	626,5
***KRAS* p.G13D**	6,30%	1596	164,7	4,50%	1410	55,7

Table S3: Sequencing performance of all FFPE diagnostic patient samples (n = 116). Seven samples (2015-022, 2015-079, 2015-098, 2015-111, 2015-130, 2015-131 and 2015-138) failed in sequencing, values of these seven samples are not included in any mean values.

For Table S3, please see the attached Excel file

Table S4: Mean total amplicon coverage of 18 samples (2015-E-006, 2015-E-007, 2015-E-008, 2015-004, 2015-005, 2015-014, 2015-015, 2015-016, 2015-017, 2015-018, 2015-020, 2015-021, 2015-083, 2015-084, 2015-093, 2015-094, 2015-096, 2015-097) from five different analyzes. Shown are the total mean amplicon coverage, as well as the forward and reverse mean coverage.

For Table S4, please see the attached Excel file

Table S5: All 269 detected variants in 185 patient samples including diagnosis, variant allele frequency, reads and quality of the variant call.

For Table S5, please see the attached Excel file

Table S6: Covered coding regions from the 23 genes which are included in the customized MDS/MPN-Panel.

	Genes	Exons
1	*ASXL1*	12
2	*BRAF*	15
3	*CALR*	9
4	*CBL*	8, 9
5	*CSF3R*	14, 17
6	*DNMT3A*	11-23
7	*EZH2*	5-8, 14-20
8	*FLT3*	14, 15, 20
9	*IDH1*	4
10	*IDH2*	4
11	*JAK2*	12, 14
12	*KIT*	8, 10, 11, 17
13	*KRAS*	2, 3
14	*NPM1*	12
15	*NRAS*	2, 3
16	*RUNX1*	3-8
17	*SETBP1*	4
18	*SF3B1*	14, 15
19	*SRSF2*	1
20	*TET2*	3-11
21	*TP53*	2-11
22	*U2AF1*	2, 6
23	*WT1*	7, 9

Table S7: Amplicon primer of the 243 amplicons of the MDS/MPN-Panel

For Table S7, please see the attached Excel file
